# Prevalence and distribution of serological characteristics of weak ABO subgroups in the Chinese population

**DOI:** 10.1016/j.htct.2024.04.127

**Published:** 2024-09-10

**Authors:** Zhongying Wang, Sha Jin, Jiewei Zheng, Chenrui Qian, Xiaohong Caib, Dong Xiang

**Affiliations:** a Blood Group Reference Laboratory, Shanghai Institute of Blood Transfusion, Shanghai Blood Center, Shanghai, China; b Transfusion Department, Ruijin Hospital, Medical School of Shanghai Jiao Tong University, Shanghai, China

**Keywords:** ABO, Subgroup, Frequency, Allele, Antigen

## Abstract

**Objectives:**

This study aimed to determine the true frequency of weak ABO subgroups and to investigate the serological characteristics of various subgroup alleles in the Chinese population.

**Methods:**

A total of 2,945,643 blood samples were collected from January 2009 to December 2017. After initial screening and re-examination using automatic blood group analyzers, all ABO-discrepant samples were confirmed by standard serological analysis and molecular detection by DNA sequencing. The true frequency of weak ABO subgroups was determined by the number of ABO subgroup donors and the missed detection rate. The ABO antigen expressions corresponding to subgroup alleles were analyzed by the agglutination intensity of red blood cells.

**Results:**

The detection rate of ABO subgroups was 0.031 % (927/2,945,643). Considering the missed detection rate (27.81 %), the true frequency of ABO subgroups in the Chinese population was 0.044 %. The three most common genetic variations among blood donors in Shanghai were *BA.04, BW.12* and *BA.02. BW.03* showed the weakest B antigen expression (6.00 ± 1.97) and *B^var-^*^1^ the strongest (9.20 ± 1.10).

**Conclusion:**

Many ABO subgroups were missed. *BA.04* was found to be the most common subgroup allele in the Chinese population. Different ABO subgroup alleles exhibit different ABO antigen expression patterns.

## Introduction

The ABO system was the first human blood group system discovered by Karl Landsteiner in 1900 ^1^ and it remains the most important and widely studied blood group system in transfusion medicine.^2^ Rare genetic variations resulting in weak ABO phenotypes, known as ‘weak ABO subgroups’, is a worldwide long-term research topic. In recent years, various subgroups with different molecular etiologies have been reported.^3^, ^4^, ^5^, ^6^, ^7^

The true frequencies of the ABO subgroup phenotypes have not been well studied due to the relatively low prevalence and the potential for missed detection. This low prevalence also obscures the relationship between the phenotype and genotype. The detection rate of the ABO subgroup in the Han Chinese population is reportedly approximately 0.015 %,^8^ yet this might be lower than the true frequency due to the lack of an estimate of the missed detection rate (MDR).

The frequencies of certain weak ABO subgroups have been reported in several populations.^9^ A_3_ is the most frequent weak A phenotype in Canadians, with a frequency of 0.0011 %.^10^ The frequency of A_x_ in France has been estimated to be 1 in 77,000 (0.003 % of group A) ^11^ and 1 in 40,000.^12^ Other A subgroups include A_m_ and A_el_, which are very rare subgroups with low frequencies and different ABO gene variations.^13^^,^^14^ Among B subgroups, B_3_ is relatively common, with a frequency of 1 in 10,000 group B French donors,^15^ 1 in 900 group B Chinese donors and 1 in 1800 group A_1_B Chinese donors.^16^ Cis-AB and B(A) phenotypes have also been frequently detected in Chinese ABO subgroup individuals, with a total frequency of 0.0083 %.^17^ In Japan, B_m_ is common (a total frequency of 0.0244 %) among B subgroups.^18^

Our previous study focused on molecular genetic analysis of ABO subgroup individuals, identification of novel subgroup alleles and elucidation of the molecular mechanisms involved.^4^^,^^19^^,^^20^ However, the distribution characteristics of these alleles and their individual differences in the antigen expression remain largely unknown.

Therefore, we systematically and comprehensively analyzed many ABO group samples, estimated the MDR of the typing process, and investigated the relative percentages (RPs) and antigen expressions of different ABO subgroup alleles. This study is the first to propose an MDR around the world and to provide a practical estimation method. The value obtained in this study is closer to the true prevalence, which is highly important as a reference in blood typing and investigation of subgroup frequency.

## Materials and methods

### Samples

Peripheral blood specimens (*n* = 2,945,643) were collected from blood donors (*n* = 2,208,563) at Shanghai Blood Center (SBC) from January 2009 to December 2017. Blood donors may donate more than once, and in this study, we called such individuals ‘repeat donors’. Weak ABO subgroup donors were blood grouped independently every time without consideration of their prior ABO results. This study was approved by the scientific ethics committee (institutional review board registration number: SBC-IRB-2017-18, SBC).

### Initial screening of ABO subgroups via the automatic microplate method

All samples were analyzed by the automatic microplate method using the automatic blood group analyzer Galileo (Immucor, Norcross, GA, USA) or the PK7300 system (Beckman Coulter, Tokyo, Japan) and the Tecan–Microplate Reader system (Tecan, Zurich, Switzerland) according to the instrument operation procedures. The following reagents were used: monoclonal anti-A (clone: F98 7C6), anti-B (clone: F84 3D6 + F97 2D6), and ABO red cells from Immucor (Norcross, GA, USA); monoclonal anti-A (clone: SRBC-B3), anti-B (clone: SRBC-C1), and ABO red cells from the Shanghai Hemo Pharmaceutical and Biological Company (SHPBC), Shanghai, China. After duplicate detection by the original method, samples with ABO discrepancies were selected for further identification.

### Serological identification of the ABO subgroups

Both forward and reverse typing were carried out by tube agglutination according to modern standard methods ^21^ and serological diagnostic classification.^9^ An absorption-elution test ^9^ was performed to identify the ‘el’-type subgroup when no antigen was detected by the tube agglutination test. The reagents used included: monoclonal anti-A (clone: SRBC-B3), anti-B (clone: SRBC-C1), anti-H (clone: H5B12), ABO red cells, A_2_ cells, and lectin anti-A_1_ from SHPBC; anti-AB (clone: 152D12 + 9113D10) from DIAGST, Loos, France; and polyclonal anti-A and anti-B (titer ≥64) manufactured by the Blood Group Reference Laboratory, Shanghai, China.

### Molecular detection of the ABO subgroup

Genomic DNA of discrepant samples was extracted using a DNA extraction kit (Tiangen, Beijing, China). The ABO gene was amplified by polymerase chain reaction (PCR). The primer sets and PCR amplification conditions used have been described previously.^19^ The ABO gene sequences of exons 6 to 7 and their boundary regions were analyzed. If no mutations previously associated with ABO subgroups were detected, the sequences of exons 1 to 5 and their splice sites, promoter region, enhancer elements, and even introns were amplified to detect possible mutations. ABO mutations and alleles were named according to the nomenclature used by International Society of Blood Transfusion (ISBT) or the original literature in brackets if ISBT names were unavailable.

### Estimating the missed detection rate and frequency of ABO subgroup blood donors

All ABO subgroup blood donors were classified according to number of donations (x) into repeat donors (*x* ≥ 2) and first-time donors (*x* = 1). Times detected and number of missed detections of each classification were counted to calculate the relative missed detection rate (RMDR) (*y* = number of missed detections/ times detected × 100). The regression line was based on the RMDR (y) and number of donations (x, *x* ≥ 2) to determine the theoretical RMDR of first-time donors (RMDR1) when *x* = 1.

The theoretical number of missed detections in first-time donors (M1) was calculated as RMDR1 × times detected of first-time donors.

The MDR of ABO subgroup blood donors was calculated as follows: MDR = (total number of missed detections of repeat donors + M1)/total times detected × 100.
The true frequency of ABO subgroup blood donors was calculated as follows: frequency = (number of first-time subgroup donors + number of repeat subgroup donors)/number of total donors × (1 + MDR).

### Statistical analysis

Statistical analysis was carried out using computer software (GraphPad Prism 6 version 6.01, GraphPad, Inc., USA).

## Results

### Detection rate of weak ABO subgroups

A total of 927 weak ABO subgroups were identified among 2,945,643 blood samples, with a detection rate of 0.031 %. The detection rates of weak AB, B and A subgroups were 0.018 % (524/2,945,643), 0.010 % (308/2,945,643) and 0.003 % (95/2,945,643), respectively. Thus, the AB subgroup was the most common weak ABO subgroup among Chinese blood donors.

### Missed detection rate and frequency of weak ABO subgroup blood donors

A total of 766 weak ABO subgroup donors were identified from among 2,208,563 blood donors, including 574 first-time donors and 192 repeat donors. The 192 repeat donors donated 634 times, of which 281 donations were not identified as having weak subgroups.

The detection and missed detection details for the subgroups of the 192 repeat blood donors are shown in Table 1.

**Figure 1:**
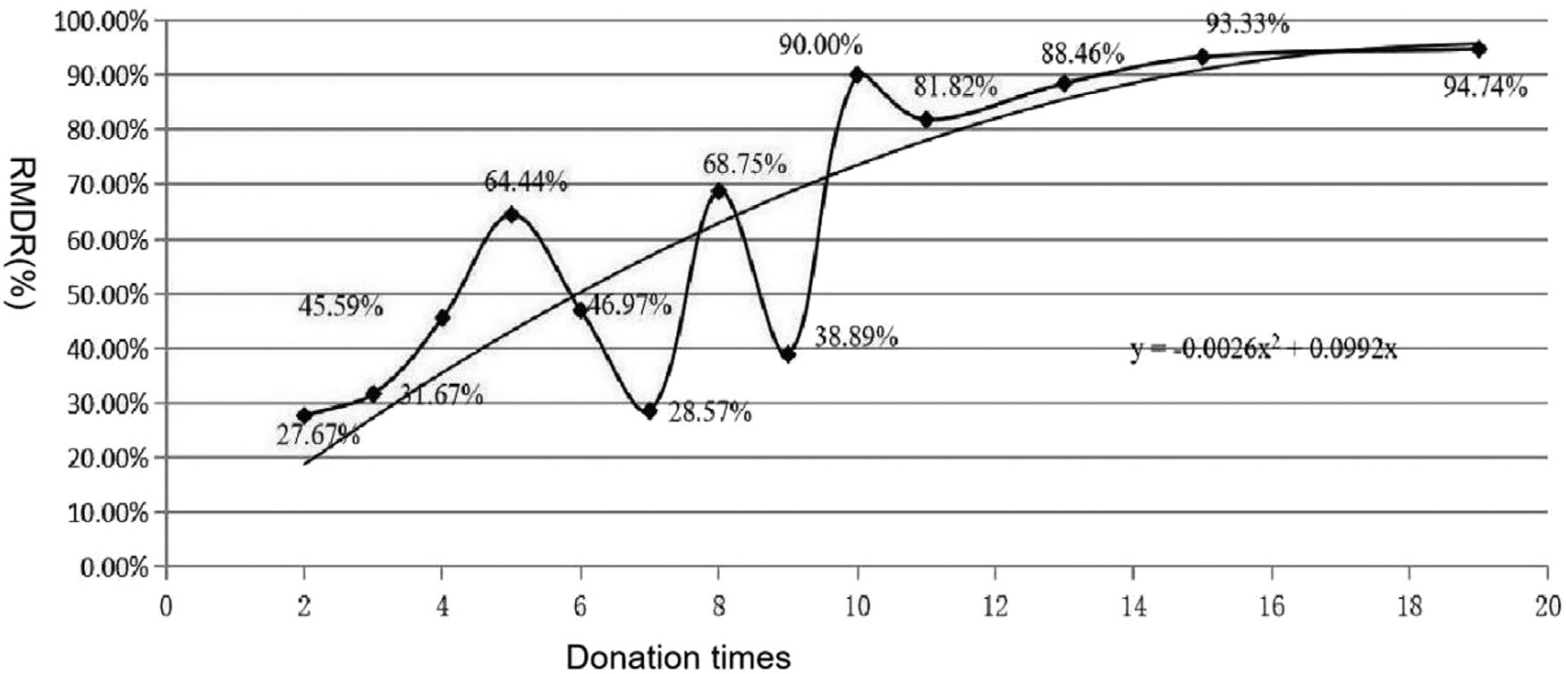
The regression line of ABO subgroup repeat blood donors. Y is the relative missed detection rate (RMDR) of ABO subgroup repeat blood donors. X is the number of donations (*x* ≥ 2).
Based on the formula: frequency = (number in the first-time donor subgroup + number in the repeat donor subgroup)/number of total donors × (1 + MDR), the frequency of ABO subgroup blood donors was (574 + 192)/2,208,563 × (1 + 27.81 %) = 0.044 %.

The regression line was *y* = −0.0026x^2^ + 0.0992x (Figure 1). When *x* = 1 and *y* = 9.66 %, the RMDR1 was 9.66 %, and the theoretical M1 = 9.66 % × 574 ≈ 55. As a result, MDR = (281 + 55)/(634 + 574) × 100 = 27.81 %.

**Table 1:** Detection and missed detection details of subgroup blood donors in the study period.

Donations times	Missed times	Donors	Detection times	Missed detection times (M)	RMDR* (%)
1	/	574	574	M1	RMDR1
2	0	46	206	57	26.67
	1	57			
3	0	17	120	28	31.67
	1	8			
	2	15			
4	0	6	68	31	45.59
	2	2			
	3	9			
5	0	1	45	29	64.44
	2	1			
	3	1			
	4	6			
6	0	2	66	31	46.97
	1	2			
	3	2			
	4	2			
	5	3			
7	0	1	14	4	28.57
	4	1			
8	5	1	16	11	68.75
	6	1			
9	1	1	18	7	38.89
	6	1			
10	9	1	10	9	90
11	9	1	9	11	81.82
13	11	1	26	23	88.46
	12	1			
15	14	1	15	14	93.33
19	18	1	19	18	94.74

RMDR: Relative missed detection rate; RMDR1: RMDR of first-time donors; M1: theoretical number of missed detections of first-time donors.

⁎ Relative missed detection rate (RMDR) = number of missed detections / number of detections × 100;.

### Missed detection rate and frequencies of different types of ABO subgroups

Detailed information on first-time donors and repeat donors for different types of ABO subgroups is shown in Table 2. According to the formula M1 = RMDR1 (9.66 %) × detection times of first-time donors, the M1s of A, B and AB subgroups were 9.66 % × 61 ≈ 6, 9.66 % × 185 ≈ 18 and 9.66 % × 328 ≈ 32, respectively.

**Table 2:** Detailed information on different types of ABO subgroup donors.

Types	First-time donors			Repeat donors			MDR* (%)	Frequency (%)
	Number	Number of detections	Theoretical number of missed detection (M1†)	Number	Number of detections	Theoretical number of missed detection (M)		
A subgroup	61	61	6	17	54	19	21.74	0.004
B subgroup	185	185	18	53	167	45	17.90	0.013
AB subgroup	328	328	32	122	413	217	33.60	0.027
Total	574	574	56	192	634	281		

MDR: Missed detection rate; RMDR1: Relative missed detection rate of first-time donors; M1: theoretical number of missed detections in first-time donors.

Frequency = (number of first-time subgroup donors + number of repeat subgroup donors)/number of total donors × (1 + MDR).

⁎ MDR = total missed detection times/total detection times × 100.

† M1 = RMDR1 × number of detections in first-time donors.

Based on the formula: MDR = total missed detection times/total detection times × 100, the MDR of the A subgroup = (19 + 6)/(54 + 61) × 100 = 21.74 %, the MDR of the B subgroup = (45 + 18)/(167 + 185) × 100 = 17.90 %, and the MDR of the AB subgroup = (217 + 32)/(413 + 328) × 100 = 33.60 %. AB subgroups were the most likely to be missed, whereas B subgroups had a low MDR.

The frequency of the A subgroup was (61 + 17)/2,208,563 × (1 + 21.74 %) = 0.004 %, the frequency of the B subgroup was (185 + 53)/2,208,563 × (1 + 17.90 %) = 0.013 %, and the frequency of the AB subgroup was (328 + 122)/2,208,563 × (1 + 33.60 %) = 0.027 %. The frequency of the AB subgroup was the highest.

### Distribution characteristics of weak ABO subgroup alleles

**Figure 2:**
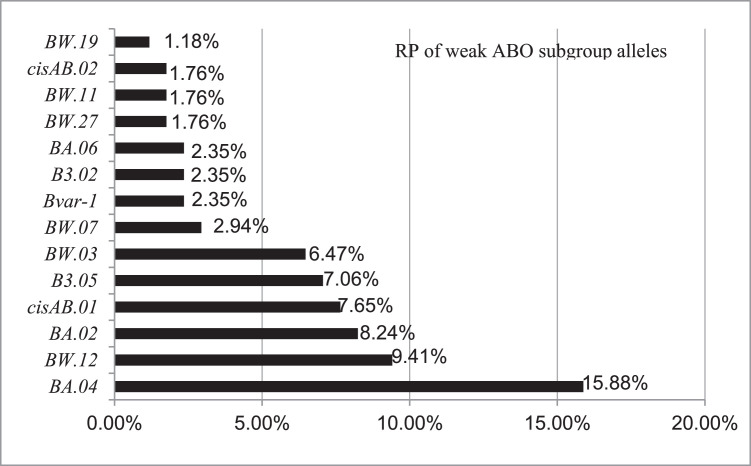
Relative percentages (RP) of weak ABO subgroup alleles in Chinese blood donors. Data were obtained for 170 different blood donors in Shanghai by Sanger sequencing. The RP of blood donors in Shanghai was calculated as the number of mutated genes per 170 × 100. For 49 other alleles, the RP was <1.18 % and is not listed.

After removing the *A2* and *Aint* alleles, typical gene results, and repeat blood donors, 170 mutated genes and >50 alleles from peripheral blood samples of blood donors were analyzed at SBC. The data indicated that *BA.04, BW.12, BA.02* and *cisAB.01* were relatively common among blood donors in Shanghai, with RPs of 15.88 %, 9.41 %, 8.24 % and 7.65 %, respectively (Figure 2).

### Discrepancy in antigen expression among different ABO subgroup alleles

According to the agglutination scores of the Association for the Advancement of Blood and Biotherapies (AABB), the serological agglutination intensities of 198 molecular detection cases were summed to assess the discrepancy in the expressions of ABO antigens. Only weak subgroup alleles that were detected in at least five individuals by serological agglutination are listed in Table 3.

**Table 3:** Agglutination intensity of monoclonal antibody reagents of different ABO subgroup alleles.

Alleles	Monoclonal anti-A (mean ± SD)	Monoclonal anti-B (mean ± SD)	Cases
ABO*BA.06	10.67 ± 1.63	12 ± 0	6
ABO*BA.04	6.45 ± 1.99	11.79 ± 0.62	47
ABO*BA.02	9.44 ± 1.23	11.76 ± 0.66	25
ABO*cisAB.01	11.88 ± 0.49	9.61 ± 1.67	31
*Bvar-1*		9.20 ± 1.10	5
ABO*BW.12		8.78 ± 1.22	18
ABO*BW.11		8.43 ± 1.81	7
ABO*B3.05		7.54 ± 1.94	13
ABO*BW.19		7.40 ± 1.34	5
ABO*BW.07		7.17 ± 2.48	6
ABO*B3.02		7.20 ± 4.38	5
ABO*BW.27		6.83 ± 1.90	12
ABO*BW.03		6.00 ± 1.97	17

SD: Standard deviation.

As shown in Table 3, the agglutination intensity of monoclonal anti-A of *BA.04* was the weakest indicating that the A antigen expression was the weakest among the four weak subgroup alleles.

Among the nine weak B alleles, *BW.03* showed the weakest agglutination of monoclonal anti-B (6.00 ± 1.97), and *B^var-1^* ^21^ had the strongest agglutination (9.20 ± 1.10). This result indicates that the B antigen expression level of *B^var-1^* was significantly greater than that of *BW.03*.

Among the six *BW* alleles, *BW.12* and *BW.11* (8.78 ± 1.22 and 8.43 ± 1.81, respectively) displayed the strongest agglutination intensities of monoclonal anti-B. This result indicates that the two *BW* alleles had the strongest antigen expressions (no significant difference).

## Discussion

This study was conducted on 2.9 million volunteer blood donors in Shanghai, which is the largest sample size to date of studies on ABO subgroups.

According to the statistical analysis of ABO subgroups of repeat blood donors, up to 27.81 % of ABO subgroups were not identified during ABO blood typing, which might indicate that the frequency reported in the literature is much lower than the true value. The MDR of the AB subgroup was 33.60 %, which was much greater than that of the A and B subgroups. Overall, the AB subgroup was more prone to missed detection than the A and B subgroups. One of the reasons might be that the H antigen of the A and B subgroups is enhanced, which is better to confirm the subgroup using the anti-H reagent, but that the H antigen of the AB subgroup is enhanced only when the allele is *O*.

Accounting for MDR, the ABO subgroup frequency of blood donors was found to be 0.044 % in Shanghai, which was much greater than previously reported.^8^ Additionally, we report the detection rates of the A, B and AB subgroups (0.003 %, 0.010 %, and 0.018 %, respectively) and obtained relatively accurate prevalence rates (0.004 %, 0.013 %, and 0.027 %, respectively) in Shanghai. Obviously, regardless of the detection rate or frequency, the AB subgroup is the most common ABO subgroup in the Chinese blood donor population. The A subgroup showed the lowest frequency in the voluntary blood donor population; the most common A subgroups, such as A2 and Aint, were excluded from in this study.

There are many reasons for the high MDR of ABO subgroups. For instance, the initial screening and serological identification of ABO subgroups are complex, and each may involve missed detection, such as interbatch differences in reagents, the limit of the automatic blood group analyzer, and individual (skill) differences among technicians. We utilized three automatic platforms. Samples (*n* = 1,874,808) were initially screened twice from January 2009 to October 2014: one by Galileo or the PK7300 system with monoclonal anti-A anti-B from Immucor; and one by the Tecan–Microplate Reader system with monoclonal anti-A and anti-B from SHPBC. The detection rate was 0.029 %. From November 2014 to December 2017, all samples (1,070,835) were screened once using one of the platforms with monoclonal anti-A and anti-B from Immucor. The detection rate was 0.035 %. Compared to that of the previous two initial screenings, the detection rate was slightly better (*p*-value >0.05). Therefore, the second screening did not increase the missed detection rate of ABO subgroups, but it indicated that there are differences in the ability of subgroup detection between different automatic platforms. In addition, the complex characteristics of ABO subgroups are one of the reasons for missed detection, such as A_el_. Due to the presence of irregular anti-A, it is likely to be mistaken as the O type by the automatic blood group analyzer. Therefore, in practical work, typing cannot rely entirely on an automatic blood group analyzer.

In general, we should employ a series of measures to minimize MDR strains. First, all microplate results should be manually reviewed during the initial screening. Second, it is necessary to establish a complete and systematic operating procedure for blood typing and strictly control the centrifuge, incubation, and environmental temperature and humidity conditions during ABO blood typing, and technicians should be regularly trained to avoid weak agglutination and missed detection caused by nonstandard operations. Third, tube agglutination should be standardized. On the basis of the AABB observation agglutination method,^22^ a shaking step was added in this study to observe the firmness of the agglutination block to avoid missing weak agglutination.

To investigate the clinical hazard of missed ABO subgroups, we traced the transfusion effects of several ABO subgroup blood samples to the transfusion departments of multiple tertiary hospitals and found no data on transfusion reactions or invalid transfusions. A possible explanation is that subgroups with strong antigen expression, such as A_3_B and cisAB, are less likely to be missed during detection, and for subgroups with weak antigen expression or weak anti-A, anti-B is not sufficient to cause a severe transfusion reaction and thus has a little impact on patients.

The ABO blood group system is clearly a critical player in the modern era of genomic medicine.^23^ *BA.04* was the most common mutated allele in Shanghai blood donors, and B alleles were the most complicated. This study identified several new ABO alleles that are being submitted. Although our understanding of the structures of A and B transferases and their enzymology has dramatically improved,^24^, ^25^, ^26^ the effects of these enzymes on the serological characteristics of different ABO subgroups have yet to be elucidated. Moreover, the relationship between serological characteristics and molecular mechanisms has received little attention. This study preliminarily evaluated the correlation between these factors using statistical methods and provided theoretical evidence for future molecular diagnoses.

The weakest B antigen expression was found for *BW.03*, with *B^var-1^* having the strongest expression. Two critical mutations in *B^var-1^* are p.Val175Met and p.Arg249Gln, which are near the four critical residues ^21^. These findings suggest that mutations near the four critical residues have weak effects on antigen expression. The critical mutation sites in *BW.03* are p.Asp302Gly and p.Arg241Trp 27. Both these sites are located in the C-terminal domain, which binds disaccharide acceptors. These findings suggest that amino acid changes in the receptor-binding site have a strong effect on antigen expression, decreasing expression of the B antigen and causing weak serological agglutination intensity, with no obvious individual differences.

This comparison revealed significant relationships among the expression level, individual differences in antigens, ABO gene mutation sites and amino acid changes, but these relationships need to be verified by additional experiments. This research involved a large sample size and long study period, and some blood donors may be related. However, due to the privacy protection of blood donors, data on the relationships between blood donors were unavailable - this was the limitation of this study.

## Conflicts of interest

We declare that we have no conflicts of interest relevant to the content of this paper.
